# The practice of defensive medicine among hospital doctors in the United Kingdom

**DOI:** 10.1186/1472-6939-14-42

**Published:** 2013-10-29

**Authors:** Osman Ortashi, Jaspal Virdee, Rudaina Hassan, Tomasz Mutrynowski, Fikri Abu-Zidan

**Affiliations:** 1 Department of Gynaecology, College of Medicine and Health Sciences, United Arab Emirates University, Al Ain, United Arab Emirates; 2 Geriatric and General Internal Medicine, London Deanery, London, UK; 3 Obstetrics and Gynaecology, Wales Deanery, Wales, UK; 4 Department of Urology, Medical University of Warsaw, Warsaw, Poland; 5 Department of Surgery and Trauma, College of Medicine and Health Sciences, United Arab Emirates University, Al Ain, United Arab Emirates

## Abstract

**Background:**

Defensive medicine is defined as a doctor’s deviation from standard practice to reduce or prevent complaints or criticism. The objectives of this study were to assess the prevalence of the practice of defensive medicine in the UK among hospital doctors and the factors affecting it.

**Methods:**

A quantitative study was designed, with a detailed seventeen point questionnaire. Defensive medicine practice was assessed and tested against four factors age, gender, specialty and grade. Three hundred hospital doctors from three UK hospitals received the questionnaire.

**Results:**

Two hundred and four (68%) out of 300 hospital doctors responded to the survey. Seventy eight percent reported practicing one form or another of defensive medicine. Ordering unnecessary tests is the commonest form of defensive medicine reported by 59% of the respondents. This is followed by unnecessary referral to other specialties (55%). While only 9% of the sampled doctors would refuse to treat high risk patients, double this number would avoid high risks procedures all together (21%). A linear regression module has shown that only senior grade was associated with less practice of defensive medicine.

**Conclusion:**

Defensive medical practice is common among the doctors who responded to the survey. Senior grade is associated with less practice of defensive medicine.

## Background

Historically, paternalism was rife within the medical profession, who were essentially self-regulated [1]. Over time, society has become more proactive and informed about available choices. It has therefore been suggested that individuals are less likely to accept at face value what is being recommended by their doctor, creating difficulties for doctors, who are not used to having their professional judgment and integrity challenged [2]. Whilst the general public is now better informed, they have also become more risk averse, often refusing to accept the usually low probability of adverse outcomes associated with medical care and interventions [3]. This encourages doctors to avoid actions that may create risk, such that they act defensively, ordering tests not on medical grounds, but to alleviate the possibility of potential complaints or litigation. At the very least, a 'more defensible’ case is created, if litigation were to occur [4]. Such an approach is compounded by the perception that courts have a tendency to rely more on data provided by investigations than on claims of experience or medical judgment [5].

Defensive medicine is defined as a doctor’s deviation from their usual behavior or that considered good practice, to reduce or prevent complaints or criticism by patients or their families [6]. The United States Congress expand this definition to include the action of ordering tests, procedures and visits, or avoidance of high risk patients or procedures with the primary (but not sole) aim, of reducing mal-practice liability [7]. A more narrow approach was adopted in Summerton’s 2000 study on defensive medical practices in General Practice; 'the ordering of treatments, tests, and procedures for the purpose of protecting the doctor from criticism rather than diagnosing or treating the patient’ [8]. Defensive medical practices can be either positive or negative. When extra tests and procedures are performed primarily to reduce malpractice liability, this is a positive defensive medicine. Negative defensive medicine consists of avoidance of certain patients and procedures, thereby withdrawing medical services, and can deny patients productive care.

For those unfortunate enough to have received a complaint or experienced litigation, the response is often deeply personal, with the affected doctor feeling anger, guilt, shame and loss of confidence, with some considering leaving the profession [9]. Although the effect of perceived litigation threat on doctors’ behavior itself is interesting, the key question to consider is whether such an effect produces a positive or negative outcome for the patient. Where doctors order diagnostic tests in the absence of indicators suggesting that these are in the patient’s best interests, patients may be exposed to risk of injury from the unnecessary and often invasive procedures, which may be greater than that of missing an unlikely diagnosis [10,11].

In a national survey carried out in the USA among neurosurgeons 96% reported practicing defensive medicine [3]. The epidemic of defensive medicine has also spread to Europe where 94% of gastroenterologists and 83% of surgeons and anesthetists in Italy reported practicing defensive medicine [12,13]. The situation is even worse in Japan as 98% of survived gastroenterologists also reported practicing at least one or another form of defensive medicine [14].

In the United Kingdom (UK), studies attempting to ascertain the prevalence of defensive practices have been extremely limited. Summerton helped shed some light on the issue in the context of General Practice in 2000 [8,15]. This observational study compared the prevalence of negative defensive medical practices in 1999 to those by the same doctors in 1994, and concluded that GPs were significantly more likely to undertake diagnostic testing, refer patients and avoid treating certain conditions at the later date. This is despite the fact that GPs are less likely to be subjected to a court action for negligence than their hospital colleagues [8].

The objectives of this study were to assess the prevalence of the practice of defensive medicine among hospitals doctors in the UK and the factors affecting it.

## Methods

A quantitative study was designed, with a detailed seventeen point questionnaire. Ethical approval was obtained from Research and Development Office of Cardiff and Vale University Local Health Board. The questionnaire was validated by experts from Law School at Cardiff University and we have been ruled by the guidelines on survey research. The questionnaire was initially drafted and subsequently modified following advice obtained during piloting. Ten doctors of three grades were interviewed for piloting; four questions were modified following the initial piloting phase. Three hospitals were chosen from two deaneries, two in South Wales and one in Kent. One of the hospitals is a University hospital and the other two were district general hospitals. The hospitals were chosen on the basis of convenience sampling. All the three hospitals are from National Health Service (NHS), none is private, however for ethical reason we could not reveal the names of the three hospitals. The study was conducted from April 2008 to March 2011. Lists of all doctors employed by the hospitals and working in all specialties were obtained from the medical staffing departments in two hospitals; doctors were emailed to complete the internet version. Paper questionnaires were distributed to doctors in the third hospital as we found difficulty in obtaining their email list. Efforts were made to ensure doctors in different departments and of differing grades were asked to participate. Three hundred doctors were approached either personally (n = 56) or by email (n = 148), overall 204 responded, making the response rate 68%. The questionnaire sought demographic information about age, gender, specialty and grade. We also tested awareness about, and personal use of different aspects of defensive medical practice.

For the purpose of this study, doctors who are practicing ANY of the following are considered practicing defensive medicine: ordering tests that are probably not clinically indicated to avoid litigation, carrying out interventions or procedures that are probably unnecessary to avoid litigation, arranging unnecessary referrals to other specialties to avoid litigation, prescribing medications to prevent later criticism or litigation, refusing to treat high risk patients to avoid the possibility of litigation stemming from complications, or avoiding high risk procedures to avoid the possibility of litigation stemming from complications.

Four factors were tested in relation to the practice of defensive medicine age, gender, specialty and grade. Doctors were divided into four age groups, 20-30, 31-40, 41-50 and more than 50 years. Specialties were categorized as Medicine, Surgery, Obstetrics and Gynecology, Pediatrics and Other specialties. The doctors’ grades were divided into three grades: juniors, middle grades and seniors. Junior grades include doctors in foundation year one and two, core trainees, and the previous senior house officer grade. Middle grade included staff grades, specialty doctors and associate specialists. Senior grade include only consultants. The outcome variable of the logistic regression model was the practice of defensive medicine while the predictors were age, gender, grade and specialty.

Univariate analysis was done using Fisher’s Exact test to compare doctors who practice defensive medicine and those who do not. Significant factors were then entered into a backward stepwise likelihood ratio logistic regression. A p value of ≤ 0.05 was considered significant. Data were analyzed with PASW Statistics 18, SPSS Inc, USA.

The required sample size was calculated to determine the prevalence of defensive medicine with an estimated prevalence of 90% with 5% bound of error and 95% level of confidence. On this basis, the necessary sample size was 139 doctors. Sample size was also calculated for consideration of associated factors and since no previous estimates were available for factors, we kept estimated prevalence for factors as 50% to achieve the OR of 1.5. With this calculation, the required sample size was 130 doctors. Hence 139 were kept as the final optimal sample size. As the questionnaire was electronic or an anonymous paper version, we inflated our sample size 40% to adjust for non-responders resulting in final sample size of around 200 doctors, as this was voluntary emailed based survey we approached 300 doctors to account for non-respondents.

## Results

Table 1 show the characteristics of the participants, 67% were 40 years old or younger. Males were 57% of the participants. Surgeons were the least represented specialty (12%). Consultants account for 47% of the respondents.

**Table 1:** Characteristics of participants

**Characteristics**	**Number**	**Percentage (%)**
**Age**		
20-30	68	33.3%
31-40	69	33.8%
41-50	41	20%
More than 50	26	12.7%
**Gender**		
Male	117	57.4%
Female	87	42.6%
**Specialty**		
Medicine	46	22.5%
Surgery	25	12.3%
O and G	48	23.5%
Pediatrics	39	19.1%
Others	46	22.5%
**Grades**		
Junior	75	36.7%
Middle	53	25.9%
Senior	76	37.4%

O & G = Obstetrics and Gynaecology.

The majority of participants, 89% (n = 182) were aware of the concept of defensive medical practice. Only 14% (n = 29) believed that they are working in a blame free culture while 86% (n = 175) believed the opposite. The majority 91% (n = 185) had the impression that legal claims against doctors are increasing and 14% (n = 29) had a direct experience of litigation. The majority of doctors had some form of indemnity cover, 90% (n = 184) [Table 2].

**Table 2:** Awareness of defensive medicine and related experience

**Awareness and experience**	**Count (n)**	**Percentage (%)**
Awareness of defensive medicine	182	89.2%
Think legal claims against doctors are increasing	185	90.6%
Has an indemnity covers (MPS / MDU / other)	184	90.2%
Direct experience of litigation (i.e. been sued)	29	14.2%
Believe in working in blame free culture	29	14.2%

Seventy eight percent (n = 159) of the surveyed hospital doctors reported practicing one or another form of defensive medicine. Those who are more than 40 years old (32%) and those who are in consultant jobs (37%) are significantly practicing less defensive medicine than others (P-value 0.001 and < 0.0001 respectively) [Table 3]. However, when we carried out backward logistic regression analysis only grade was found to affect the practice of defensive medicine (odd ratio 0.44) [Table 4].

**Table 3:** The practice of defensive medicine among the sampled doctors

**Variable**	**Defensive medicine**	**No defensive medicine**	**P-value**
	**n = 159 (%)**	**n = 45 (%)**	
**Age group**			**<0.001**
20-30	61 (38.4%)	7 (15.6%)	
31-40	56 (35.2%)	13 (28.9%)	
41-50	27 (17%)	14 (31.1%)	
More than 50	15 (9.4%)	11 (24.4%)	
**Gender**			**0.31**
Male	88 (55.3%)	29 (64.4%)	
Female	71 (44.7%)	16 (35.6%)	
**Grade**			**<0.0001**
Junior	67 (42.1%)	10 (22.2%)	
Middle grade	47 (29.6%)	6 (13.3%)	
Consultant	45 (28.3%)	29 (64.5%)	
**Specialty**			**0.32**
Medicine	37 (23.3%)	9 (20%)	
Surgery	20 (12.6%)	4 (8.9%)	
O & G	42 (26.4%)	7 (15.6%)	
Pediatrics	27 (16.9%)	12 (26.7%)	
Others	33 (20.8%)	13 (28.8%)	

Data presented as numbers (%).

O & G = Obstetrics and Gynaecology.

P-value = Fisher’s exact test.

**Table 4:** Backward logistic regression model defining factors affecting the practice of defensive medicine

**Variable**	**Estimate**	**SE**	**Wald test**	**p value**	**Odds ratio**
Grade	0.81	0.22	13.76	< 0.0001	0.44
Constant	3.01	0.54	31.53	< 0.0001	20.3

Ordering un-necessary tests was the most common form of defensive medicine practiced by the sampled hospital doctors (59%) followed by arranging un-necessary referral to other specialties (55%). Nine percent would refuse to treat high risk patients. However, over the double (21%) would avoid high risks procedures all together [Table 5]. The logistic regression model was highly significant (p < 0.0001, R square = 0.1).

**Table 5:** Different forms of defensive medicine practiced by the respondents (n = 204)

**Practice**	**(n)**	**Percentage (%)**
Ordering tests un-necessary tests	121	59.3%
Unnecessary referral	112	54.9%
Performing unnecessary intervention / procedure	56	27.5%
Prescribing un-necessary medication	47	23%
Avoiding high risk procedures	42	20.6%
Refusal to treat a high risk patients	19	9.3%

## Discussion

Two hundred and two doctors responded to our questionnaire. The response rate for this survey was good (68%), we think the factors that improved the response rate were the fact that most of respondents found the survey subject interesting and very close to their hearts, they felt that the wellbeing of doctors is often an under researched part of the care system. Secondly as we were expected weak response to the electronic version, we monitored the responses on regular bases and those who did not respond to the email survey were identified and contacted in person for face to face interview.

Most of the sampled hospital doctors were aware of the defensive medicine concept and practice (98%) on direct questioning. The NHS in the UK has been working hard for many years to create a blame free culture however our results highlighted that this far from being reality, with 86% of the doctors in this study believing they are not working in such culture. This might be due to fear of litigation among all doctors. The majority of respondents (90%) reported that they have indemnity cover though from January 1990 health authorities took over financial responsibility for negligence attributable to medical and dental staff of the hospital and community health services, as a result, it should no longer be a contractual requirement for NHS employed doctors to hold indemnity insurance for work undertaken as part of their employment contract. However separate indemnity through the defense societies or other insurer must be taken out by the doctor for any work which is not covered by the indemnity scheme. Fourteen percent of all respondents and one in three consultants had a direct experience of litigation, this include any form of court process even if the doctor did not attend the court by him or herself.

More than three quarters (78%) of doctors reported practicing one form or another of defensive medicine, and although this seems to be a high prevalence rate, in fact this is well below the prevalence of defensive medicine reported in United states of America (USA) and Japan where the prevalence of defensive medicine practice is reported to be above 90% [14,16,17].

The practice of defensive medicine was found to be statistically significant less in those above the age of 40 years compare with those less than 40 years (P-value = 0.001) and those on consultant posts compared with those on other grades (P-value 0.000). However when we carried out backward logistic regression analysis only senior grade (consultant) was found to be associated with less practice of defensive medicine, in fact the practice of defensive medicine in this study tends to double as you go down from consultant grade through middle grade to junior grade (odd ratio 0.44). This suggest age is probably is not the best indicator of competency or experience, those on consultant grade seem more confident in their skills and practicing less defensive medicine. Marin and his colleagues carried out a survey among hospital doctors in Wales in 1989 and found that age and seniority are associated with more conservative and defensive practice [18].

Though one in three consultants have a direct experience with litigation, however consultants are practicing less defensive medicine. We think this is expected finding, as consultants are taking the ultimate responsibility in the NHS, most of the claims are not successful so most of the consultants are used to deal with these claims on frequent base.

The NHS in the UK has started recently moving from consultant led care to consultant delivered care; this was in response to major service reviews which indicated that public expects from NHS this type of care. However these changes face many challenges including the high cost of consultant posts expansion. This study suggested that the initial costs might be reversed later as consultants are expected to practice less defensive medicine saving money which can be used in the increasing the number of consultants.

Many other studies suggested that the risk of litigation is related to a large extent to the specialty [14], our study did not show any significant different in the practice of defensive medicine among different specialties (P-value = 0.32), however given the small sample size this is might not be a true reflection. We also found no correlation between gender and the practice of defensive medicine. (P-value = 0.31).

Over half of the sampled doctors in this study (59%) practice defensive medicine in the form of ordering un-necessary tests. Interestingly this is exactly the same percentage that Nicholas Summerton found in 1995 among general practitioners in the UK where 59% said they would request diagnostic tests to avoid complaints and litigation [15]. Imaging studies were shown in some other studies to be the most common test ordered defensively and in some specialties like orthopedic surgery expensive imaging modalities such as Magnetic Resonance Imaging (MRI) represented 48.7% of the tests ordered defensively [19]. In another survey, Massachusetts physicians stated that between 20% and 30% of plain film x-rays, CT scans, MRI studies and ultrasound studies were ordered primarily for defensive purposes [20]. Ennis et al. conducted a survey among members and fellows of the Royal College of Obstetricians and gynecologists in 1991 and found that most of the surveyed doctors were using some of tests which were known to them as less accurate. The most frequent explanations given for this paradoxical finding were that such tests were an aid to clinical judgment and were necessary for medicolegal reasons. However this is not different among different grades [21].

Unnecessary referral to other specialties is also very common. In this study 55% of the sampled doctors stated that they practice this form of defensive behaviour. This is slightly less than the prevalence found by Nicholas Summerton in 1995 where 65% of GPs said that they arranged unnecessary referrals to avoid litigation [15]. In our study 27% of doctors said that they will perform unnecessary interventions or procedures to avoid the risk of litigation, this is similar to what was reported from Italy where 31% of the procedures performed by gastroenterologists were reported as defensive procedure without solid medical indication [12]. Prescribing medications to avoid the risk of litigation is also not uncommon practice among the studied hospital doctors (23%). Summerton in 1995 [15] found that 29% of GPs reported prescribing unnecessary medications to avoid the risk of litigation, whilst on his follow up study in 2000 this dropped to 21% [8], which is a similar figure to that found in this study. This form of practice can cause significant harm to patients; moreover this can cost a lot of money and increase significantly the health care bill.

While only 9% of the sampled hospital doctors would refuse to treat high risk patients on direct questioning, over double this number would avoid high risks procedures all together (21%). This is not surprising as other studies in the USA showed that up to 42% doctors working in high risk specialties like orthopedics reported that they had taken steps to restrict their practice in the previous years, including eliminating procedures prone to complications, such as trauma surgery, and avoiding patients who had complex medical problems or were perceived as litigious [16].

The cost of defensive medical practice is difficult to estimate due to the many conflicting and overlapping factors. While there have been attempts to estimate the cost of litigation and malpractice on the total health budget [22,23], only a few studies assessed the cost of defensive medical practice on heath system budget specifically. It is expected that the cost of defensive medicine is huge, in the USA it is estimated that the national cost of defensive medicine for the specialty of orthopedic surgery is $2 billion annually [24]. As 78% of the sampled doctors reported practicing one or other forms of defensive medicine we suspect from this study that the cost of defensive medical practice among hospital doctors in NHS will be very high and might be one of the major causes of the NHS budget deficits over the last decade despite the progressive increase in budget. Given the small size of this study further cost analysis studies is urgently needed to establish the overall cost of the practice of defensive medicine on the NHS budget.

Our study main limitations are the small sample size and it was carried out in only three hospitals. The use of convenient sampling and mixed technique of data collection was another limitation. As we tried to keep the questionnaire short but informative we could not increase the study validation by cloaking the questions with other topics and repeating questions phrased in a different manner. We selected the predictors of the outcome variables of the logistic regression model depending on previous studies [16,18]. The R square of our model was only 0.1. This implies that the studied significant factor explains only 10% of the variation of the data. We could have added other important predictor variables but these were personal data and would not be approved by ethical committee.

## Conclusion

Defensive medicine practice is common among hospital doctors who responded to our survey. Ordering un-necessary tests is the commonest form of the defensive medicine identified in this study. Senior grade is significantly associated with less practice of defensive medicine. Further research is needed on the cost of defensive medicine on the NHS.

## Competing interests

We certify that we have no competing interests with any organization regarding the material Discussed in this manuscript.

## Authors’ contributions

OO, JV, RH and TM participated in the design of the study, data collection, results interpretation and writing up the manuscript. FA carried out the statistical analysis, results interpretation and critically read the manuscript. All authors read and approved the final manuscript.

## Pre-publication history

The pre-publication history for this paper can be accessed here:

http://www.biomedcentral.com/1472-6939/14/42/prepub

## References

[B1] Thorpe KE The medical malpractice 'crisis’: recent trends and the impact of state tort reforms 2004 Health Aff (Millwood) Suppl Web Exclusives: W4-20-30 10.1377/hlthaff.w4.20 15452009

[B2] Nguyen Thi PL Briançon S Empereur F Guillemin F Factors determining inpatient satisfaction with care Soc Sci Med 2002 54 4 493 504 10.1016/S0277-9536(01)00045-4 11848270

[B3] Sethi MK Obremskey WT Natividad H Mir HR Jahangir AA Incidence and costs of defensive medicine among orthopedic surgeons in the United States: a national survey study Am J Orthop (Belle Mead NJ) 2012 41 2 69 73 22482090

[B4] Brilla R Evers S Deutschlander A Are neurology residents in the United States being taught defensive medicine Clin Neurol Neurosurg 2006 108 4 374 377 10.1016/j.clineuro.2005.05.013 16040189

[B5] Chen XY Defensive medicine or economically motivated corruption? A Confucian reflection on physician care in China today J Med Philos 2007 32 6 635 648 10.1080/03605310701681021 18027252

[B6] Toker A Shvarts S Perry ZH Clinical guidelines, defensive medicine, and the physician between the two Am J Otolaryngol 2004 25 4 245 250 10.1016/j.amjoto.2004.02.002 15239030

[B7] Corrigan J Wagner J Wolfe L Report from congress medical malpractice reform and defensive medicine Cancer Invest 1996 14 3 277 284 10.3109/07357909609012149 8630689

[B8] Summerton N Trends in negative defensive medicine within general practice Br J Gen Pract 2000 50 565 566 10954939 PMC1313753

[B9] Vincent C Bark P Jones A The impact of litigation on obstetricians and gynaecologists J Obstet Gynaecol 1994 14 381 387 10.3109/01443619409027617

[B10] Dubay L Kaestner R Waidmann T The impact of malpractice fears on cesarean section rates J Health Econ 1999 18 4 491 522 10.1016/S0167-6296(99)00004-1 10539619

[B11] Sharzer S Defensive medicine is worthless–on two counts Med Econ 1993 70 6 41 42 44-5, 48 10171389

[B12] Elli L Tenca A Soncini M Spinzi G Buscarini E Conte D Defensive medicine practices among gastroenterologists in Lombardy: between lawsuits and the economic crisis Dig Liver Dis 2013 45 6 469 473 doi:10.1016/j.dld.2013.01.004 10.1016/j.dld.2013.01.004 23402738

[B13] Catino M Celotti S The problem of defensive medicine: two Italian surveys Stud Health Technol Inform 2009 148 206 221 19745252

[B14] Hiyama T Yoshihara M Tanaka S Urabe Y Ikegami Y Fukuhara T Chayama K Defensive medicine practices among gastroenterologists in Japan World J Gastroenterol 2006 12 47 7671 7675 17171798 10.3748/wjg.v12.i47.7671 PMC4088051

[B15] Summerton N Positive and negative factors in defensive medicine: a questionnaire study of general practitioners BMJ 1995 310 6971 27 29 10.1136/bmj.310.6971.27 7827550 PMC2548439

[B16] Studdert DM Mello MM Sage WM DesRoches CM Peugh J Zapert K Brennan TA Defensive medicine among high-risk specialist physicians in a volatile malpractice environment JAMA 2005 1;293 21 2609 2617 10.1001/jama.293.21.2609 15928282

[B17] Rodriguez RM Anglin D Hankin A Hayden SR Phelps M McCollough L Hendey GW A longitudinal study of emergency medicine residents’ malpractice fear and defensive medicine Acad Emerg Med 2007 14 6 569 573 Epub 2007 Apr 19 10.1111/j.1553-2712.2007.tb01834.x 17446194

[B18] Marin PP Bayer AJ Tomlinson A Pathy MS Attitudes of hospital doctors in Wales to use of intravenous fluids and antibiotics in the terminally ill Postgrad Med J 1989 65 767 650 10.1136/pgmj.65.767.650 2608596 PMC2429185

[B19] Miller RA Sampson NR Flynn JM The prevalence of defensive orthopaedic imaging: a prospective practice audit in Pennsylvania J Bone Joint Surg Am 2012 94 3 e18 22298064 10.2106/JBJS.K.00646

[B20] Massachusetts Medical Society Investigation of defensive medicine in Massachusetts Available at: http://www.massmed.org//am/template.cfm?section=home6. Accessed January 11, 2010; 2008

[B21] Ennis M Clark A Grudzinskas JG Change in obstetric practice in response to fear of litigation in the British Isles Lancet 1991 338 8767 616 618 10.1016/0140-6736(91)90616-W 1679160

[B22] Kessler DP Evaluating the medical malpractice system and options for reform J Econ Perspect 2011 25 2 93 110 10.1257/jep.25.2.93 21595327 PMC3195420

[B23] NHS compensation fund gets 185m pound bailout as claims rise by 30% in a year BMJ 2012 344 e411 doi:10.1136/bmj.e411 10.1136/bmj.e411 22250222

[B24] Sethi MK Obremskey WT Natividad H Mir HR Jahangir AA Incidence and costs of defensive medicine among orthopedic surgeons in the United States: a national survey study Am J Orthop 2012 41 2 69 73 22482090

